# Association between long-term exposure to air pollutants and cardiopulmonary mortality rates in South Korea

**DOI:** 10.1186/s12889-020-09521-8

**Published:** 2020-09-14

**Authors:** Jeongeun Hwang, Jinhee Kwon, Hahn Yi, Hyun-Jin Bae, Miso Jang, Namkug Kim

**Affiliations:** 1 Department of Medicine, University of Ulsan College of Medicine, Asan Medical Center, Seoul, Republic of Korea; 2 Department of Biomedical Engineering, Asan Medical Institute of Convergence Science and Technology, University of Ulsan College of Medicine, Asan Medical Center, Seoul, Republic of Korea; 3 Asan Institute for Life Sciences, Asan Medical Center, Seoul, Republic of Korea; 4 Department of Convergence Medicine, University of Ulsan College of Medicine, Asan Medical Center, 88, Olympic-ro 43-gil, Songpa-gu, Seoul, 05505 South Korea; 5 Department of Radiology, University of Ulsan College of Medicine. Asan Medical Center, Seoul, Republic of Korea

**Keywords:** Ischemic heart disease, Cerebrovascular disease, Pneumonia, Chronic lower respiratory disease, Air pollution, Long-term exposure, Mortality

## Abstract

**Background:**

The association between long-term exposure to air pollutants, including nitrogen dioxide (NO_2_), carbon monoxide (CO), sulfur dioxide (SO_2_), ozone (O_3_), and particulate matter 10 μm or less in diameter (PM_10_), and mortality by ischemic heart disease (IHD), cerebrovascular disease (CVD), pneumonia (PN), and chronic lower respiratory disease (CLRD) is unclear. We investigated whether living in an administrative district with heavy air pollution is associated with an increased risk of mortality by the diseases through an ecological study using South Korean administrative data over 19 years.

**Methods:**

A total of 249 Si-Gun-Gus, unit of administrative districts in South Korea were studied. In each district, the daily concentrations of CO, SO_2_, NO_2_, O_3_, and PM_10_ were averaged over 19 years (2001–2018). Age-adjusted mortality rates by IHD, CVD, PN and CLRD for each district were averaged for the same study period. Multivariate beta-regression analysis was performed to estimate the associations between air pollutant concentrations and mortality rates, after adjusting for confounding factors including altitude, population density, higher education rate, smoking rate, obesity rate, and gross regional domestic product per capita. Associations were also estimated for two subgrouping schema: Capital and non-Capital areas (77:172 districts) and urban and rural areas (168:81 districts).

**Results:**

For IHD, higher SO_2_ concentrations were significantly associated with a higher mortality rate, whereas other air pollutants had null associations. For CVD, SO_2_ and PM_10_ concentrations were significantly associated with a higher mortality rate. For PN, O_3_ concentrations had significant positive associations with a higher mortality rate, while SO_2_, NO_2_, and PM_10_ concentrations had significant negative associations. For CLRD, O_3_ concentrations were associated with an increased mortality rate, while CO, NO_2_, and PM_10_ concentrations had negative associations. In the subgroup analysis, positive associations between SO_2_ concentrations and IHD mortality were consistently observed in all subgroups, while other pollutant-disease pairs showed null, or mixed associations.

**Conclusion:**

Long-term exposure to high SO_2_ concentration was significantly and consistently associated with a high mortality rate nationwide and in Capital and non-Capital areas, and in urban and rural areas. Associations between other air pollutants and disease-related mortalities need to be investigated in further studies.

## Background

There is increasing evidence on the harmful associations between air pollution and cardiopulmonary mortality [1–9]. Many short-term studies have reported compelling evidence on such associations [2, 3, 5, 6, 9]; however, relatively limited number of long-term studies were performed. This may be partly because collecting and analyzing long-term air pollution and cardiopulmonary mortality data together are relatively difficult than collecting and analyzing short-term data together.

Although a meta-analysis by Vodonos et al. [8], and a recent large representative cohort study by Pope et al. [4] provide compelling evidence on long-term associations between air pollution and cardiopulmonary mortality in a cohort design, these studies have only focused on exposure to fine particulate matter.

However, long-term association studies on cardiopulmonary mortality performed in South Korea [10–12] have focused on particulate matter 10 μm or less in diameter (PM_10_). Kim et al. [11] used the National Health Insurance Service sample cohort representing the general population in South Korea and estimated the individual exposure to PM_10_ as a 5-year average (2002–2006); they found positive but insignificant associations between PM_10_ exposure and cardiopulmonary diseases. Tran et al. [12] found associations between pneumonia mortality and PM_10_ concentrations (2005–2015), and Kim et al. [10] reported the cardiopulmonary mortality benefits of PM_10_ reduction. Both studies were conducted in 25 districts in Seoul, the capital of South Korea.

However, there is a knowledge gap regarding whether exposure to higher concentrations of air pollutants, including carbon monoxide (CO), sulfur dioxide (SO_2_), nitrogen dioxide (NO_2_), ozone (O_3_), and PM_10_ in a residential district in South Korea over a long term, such as 19 years, would be associated with higher cardiopulmonary mortality. We investigated a total of 249 districts in South Korea from 2001 to 2018 to evaluate the associations between air pollutants including CO, SO_2_, NO_2_, O_3_, and PM_10_, and age-adjusted mortality rates related to ischemic heart disease (IHD), cerebrovascular disease (CVD), pneumonia (PN), and chronic lower respiratory disease (CLRD) nationwide after adjusting for altitude, population density, higher education rate, smoking rate, obesity rate, and gross regional domestic product per capita (GRDP). Because there may be uncaptured socioeconomic or cultural differences between the capital and non-capital areas, and urban and rural areas, we also investigated whether the associations found in the nationwide setting remained qualitatively similar in subgroups.

## Methods

### Study design and ethics

The study used an ecological design. Ethical approval was not required because the study used only publicly accessible, national statistics database.

### Air pollution

CO, SO_2_, NO_2_, O_3_, PM_10_, and PM_2.5_ concentrations measured by the National Ambient Air Quality Monitoring Information System, are publicly accessible via the AirKorea website. In South Korea, there are 332 measurement stations. Due to a shortage in measurement stations before 2015, PM_2.5_ concentrations were not assessed in the current study. The average concentrations of each pollutant per day were collected for each station. The air pollution measurement station system was not directly matched to the Si-Gun-Gu district system, on which populations and mortality statistics dataset were based. Longitudes and latitudes of all air pollution measurement stations and all districts’ administrative authorities offices were obtained, and then the average air pollutant concentrations throughout the study period for each administrative office were estimated by linearly interpolating air pollutant measurements from the nearest three stations. Python programming language version 2.7 (Python Software Foundation, Beaverton, Oregon, United States) was used in this procedure. For each air pollutant and district, the average air pollutant concentrations throughout the study period (2001–2018) was computed. The above mentioned method is largely similar to that used in our previous study [13].

### Mortality statistics

According to the 10th revision of the International Classification of Diseases, age-adjusted mortality rates were obtained from death certificates and population census data were obtained from the Korean Statistical Information Service (KOSIS) during the study period (2001–2018). In detail, mortality rates of IHD (I20–I25), CVD (I60–I69), PN (J12–J18), and CLRD (J40–J47) were obtained. As of 2018, there were 250 Si-Gun-Gus in South Korea as of 2018. Si-Gun-Gu is a level in the Korean administrative area system, which is comparable to counties in the United States. All Si-Gun-Gus in South Korea were included in this study, except Sejong-Si, which was newly designated in 2012. The mortality rates per 100,000 were age-adjusted by using the standard population as of July 1, 2010 in South Korea. The age-adjusted mortality rates were extracted from the KOSIS database and calculated as follows:

$$ \mathrm{Age}-\mathrm{adjusted}\ \mathrm{mortality}\ \mathrm{rate}=\sum \frac{\ \mathrm{mortality}\ \mathrm{rate}\ \mathrm{in}\ \mathrm{age}\ \mathrm{group}\times \mathrm{population}\ \mathrm{of}\ \mathrm{age}\ \mathrm{group}\ }{\mathrm{total}\ \mathrm{population}} $$

### Confounding factors

The annual average of confounding factors including altitude, smoking rate (rate of current smokers adjusted for the age of the national standard population), higher education rate (rate of > 15-year-old persons with college education or more in the district), obesity rate (rate of persons with body mass index > 25 kg/m^2^), population density based on the 2010 Census, and gross regional domestic production (GRDP) per capita as of 2011 were accessed for all districts using the KOSIS.

### Statistical analysis

Data are presented as median, interquartile range, and 95% confidence interval (95% CI) where applicable. Per interquartile increase of air pollutant concentrations, multivariable beta regression [14, 15] models were built, and the odds ratio of each air pollutant to the mortality rates were estimated while adjusting for the confounding factors. A basic bootstrap method was utilized to estimate the 95% CIs for the odds ratios. Statistical analyses were performed by using R statistics software version 3.6.3 (R Foundation for Statistical Computing, Vienna, Austria).

### Subgrouping schema

Two subgrouping schemas were applied: capital and non-capital areas and urban and rural areas. Among the 249 districts, the capital area included 77 districts in Seoul, the capital city of South Korea, Incheon, and Gyeonggi-do. These 77 districts are geographically in the vicinity of the capital and linked to each other by public transportation such as the subway system. The capital area contains 49% of the total South Korean population. The non-Capital area consists of the remaining 172 districts. The urban subgroup contained 168 districts identified as Gu or Si, whereas the rural subgroup contained 81 districts identified as Gun.

## Results

Table 1 shows the medians and interquartile ranges of the mortality rates of the four diseases, concentrations of the five air pollutants, and confounding factors averaged from 2001 to 2018. The population of South Korea, as of 2010 (in the middle of the study period) was 50,515,666 persons. Throughout the study period, a total of 4,558,640 all-cause mortalities were recorded. Among them, 242,711 deaths were attributed to IHD, 509,740 deaths to CVD, 160,174 deaths to PN, and 138,271 deaths to CLRD.

**Table 1:** Characteristics of the study area

Characteristics	Median (first–third quartile range)
Number of districts analyzed	249
IHD^a^: Age-adjusted mortality rate (per 100,000)	21.6 (19.4–25.3)
CVD^b^: Age-adjusted mortality rate (per 100,000)	50.7 (46.5–54.5)
PN^c^: Age-adjusted mortality rate (per 100,000)	12.2 (10.9–13.5)
CLRD^d^: Age-adjusted mortality rate (per 100,000)	13.8 (11.7–16.0)
Carbon monoxide (ppb) ^e^	554 (476–624)
Nitrogen dioxide (ppb)	20.1 (14.6–26.8)
Sulfate dioxide (ppb)	4.89 (4.15–5.56)
Ozone (ppb)	24.3 (21.6–27.6)
PM_10_ (μg/m^3^)	50.2 (46.1–55.5)
Altitude (m)	124 (59.9–220)
Population density (per km^2^)	361 (109–6042)
Higher-education rate ^f^ (%)	34.4 (22.9–43.0)
Smoking rate ^g^ (%)	25.1 (23.5–26.8)
Obesity rate ^h^ (%)	22.4 (20.9–24.4)
GRDP^i^ (million won)	22.9 (16.7–29.0)

From 2001 to 2018, the population of all 249 South Korean administrative districts, Si-Gun-Gus, were studied. The South Korean population, as of 2010 (in the middle of the study period) was 50,515,666 persons. Throughout the study period, a total of 4,558,640 all-cause deaths were recorded. Among them, 242,711 deaths were attributed to IHD, 509,740 deaths to CVD, 160,174 deaths to PN, and 138,271 deaths to CLRD

^a^Ischemic Heart Disease (IHD): Total number of deaths by IHD in 2001–2018 was 242,711. The annual raw mortality rates throughout the study period were adjusted for each district’s age distribution to the standard South Korean population in 2010

^b^Cerebrovascular Disease (CVD): Total number of deaths by CVD in 2001–2018 was 509,740

^c^Pneumonia (PN): Total number of deaths by PN in 2001–2018 was 160,174

^d^Chronic Lower Respiratory Disease (CLRD): Total number of deaths by CLRD in 2001–2018 was 138,271

^e^Air pollution data from 2001 to 2018, in daily mean concentrations according to the positions of monitoring stations were accessed using the AirKorea database. An interpolation model based on a geographical information system was applied to yield the average air pollutant concentration throughout the study period of the corresponding districts

^f^Rate of > 15-year-old persons with college education or more in 2010

^g^Rate of current smokers adjusted for the age of the national standard population in 2010

^h^Rate of population with body mass index > 25 kg/m^2^ adjusted for the age of the national standard population in 2010

^i^Gross Regional Domestic Product per capita (GRDP) in 2011

**Fig. 1:**
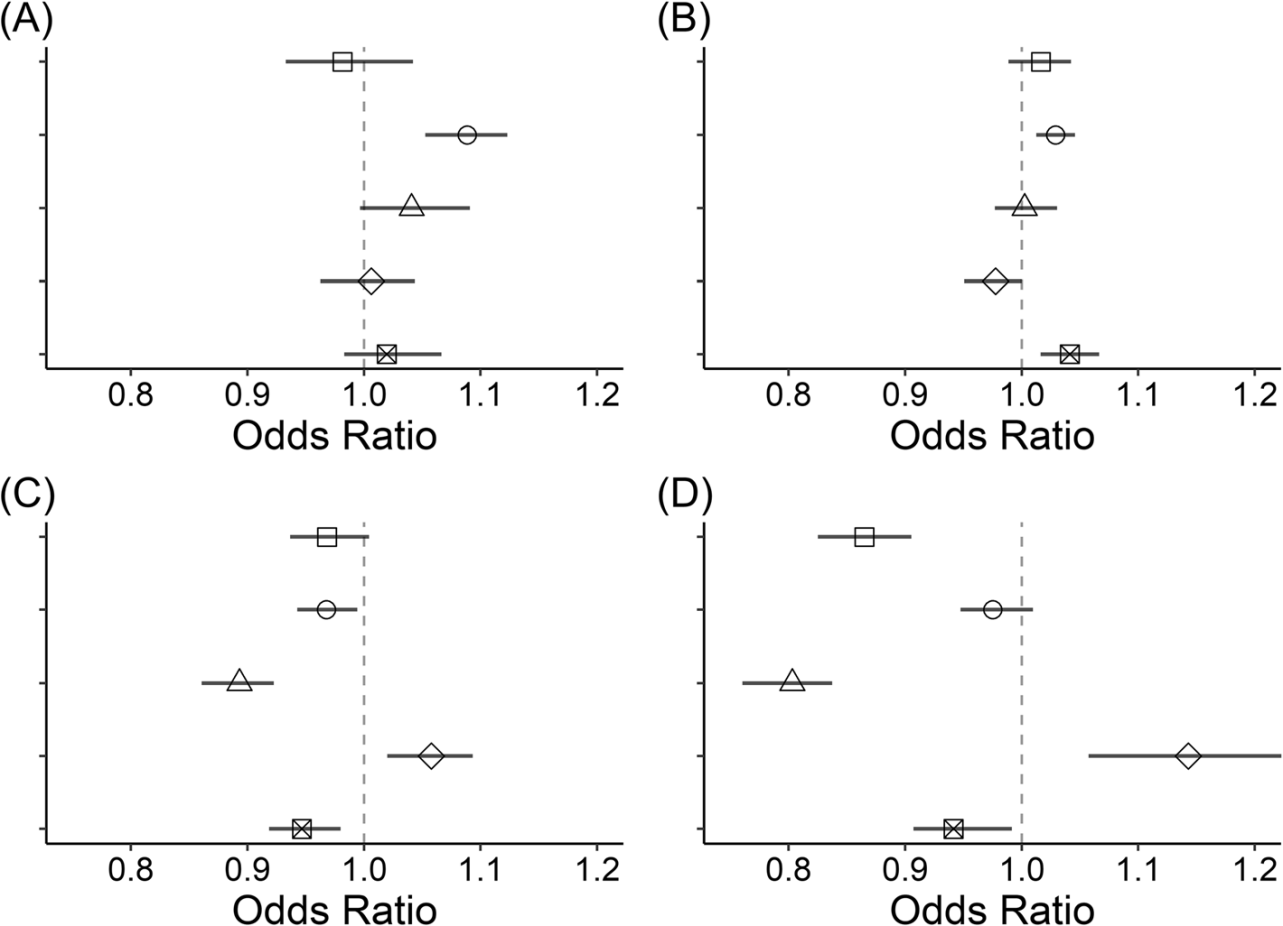
Associations between air pollutant concentrations (□: CO, ○: SO_2_, △: NO_2_, ◇: O_3_, ⊠: PM_10_) and **a** ischemic heart disease (IHD), **b** cerebrovascular disease (CVD), **c** pneumonia (PN), and **d** chronic lower respiratory disease (CLRD) mortality rates

For IHD, an increased SO_2_ concentrations were significantly associated with a higher mortality rate (odds ratio per interquartile range [OR] 1.09; 95% CI, 1.05–1.12), whereas other air pollutants had null associations. For CVD, SO_2_ (OR 1.03; 95% CI 1.01–1.05) and PM_10_ (OR 1.04; 95% CI 1.02–1.07) concentrations had significant associations with a higher mortality rate. For PN, O_3_ (OR 1.06; 95% CI 1.02–1.09) concentrations had significant positive associations with a higher mortality rate, while SO_2_ (OR 0.968; 95% CI 0.943–0.994), NO_2_ (OR 0.893; 95% CI 0.861–0.923), and PM_10_ (OR 0.947; 95% CI 0.919–0.980) concentrations had significant negative associations. For CLRD, O_3_ concentrations were associated with an increased mortality rate (OR 1.08; 95% CI 1.01–1.13), while CO (OR 0.891; 95% CI 0.856–0.935), NO_2_ (OR 0.822; 95% CI 0.780–0.865), and PM_10_ (OR 0.934; 95% CI 0.902–0.977) concentrations had negative associations. Figure 1 shows the ORs and 95% CIs of the estimated associations.

**Fig. 2:**
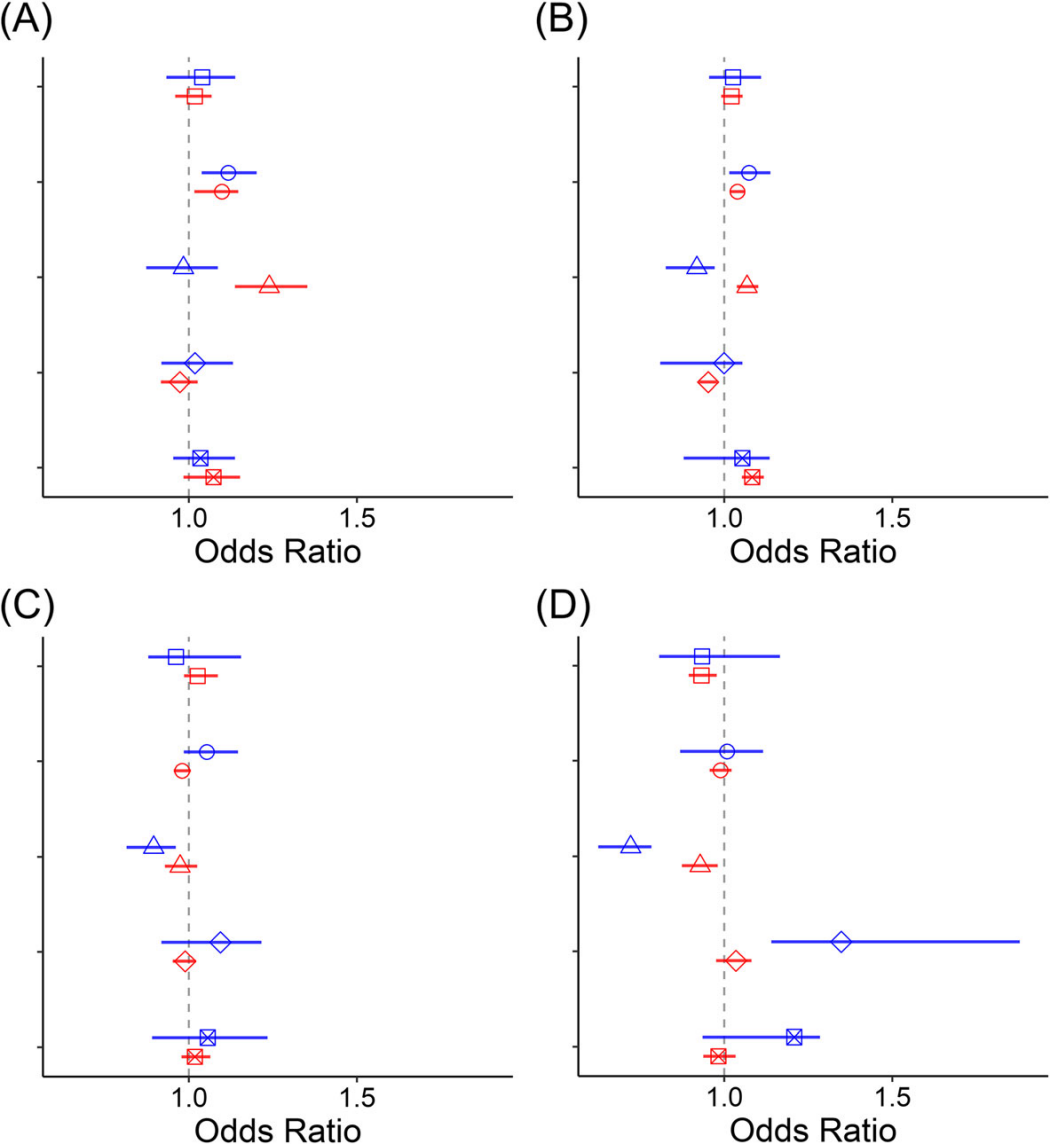
Associations between air pollutant concentrations (□: CO, ○: SO_2_, △: NO_2_, ◇: O_3_, ⊠: PM_10_) and **a** ischemic heart disease (IHD), **b** cerebrovascular disease (CVD), **c** pneumonia (PN), and **d** chronic lower respiratory disease (CLRD) mortality rates in the capital (blue) or non-capital (red) areas

**Fig. 3:**
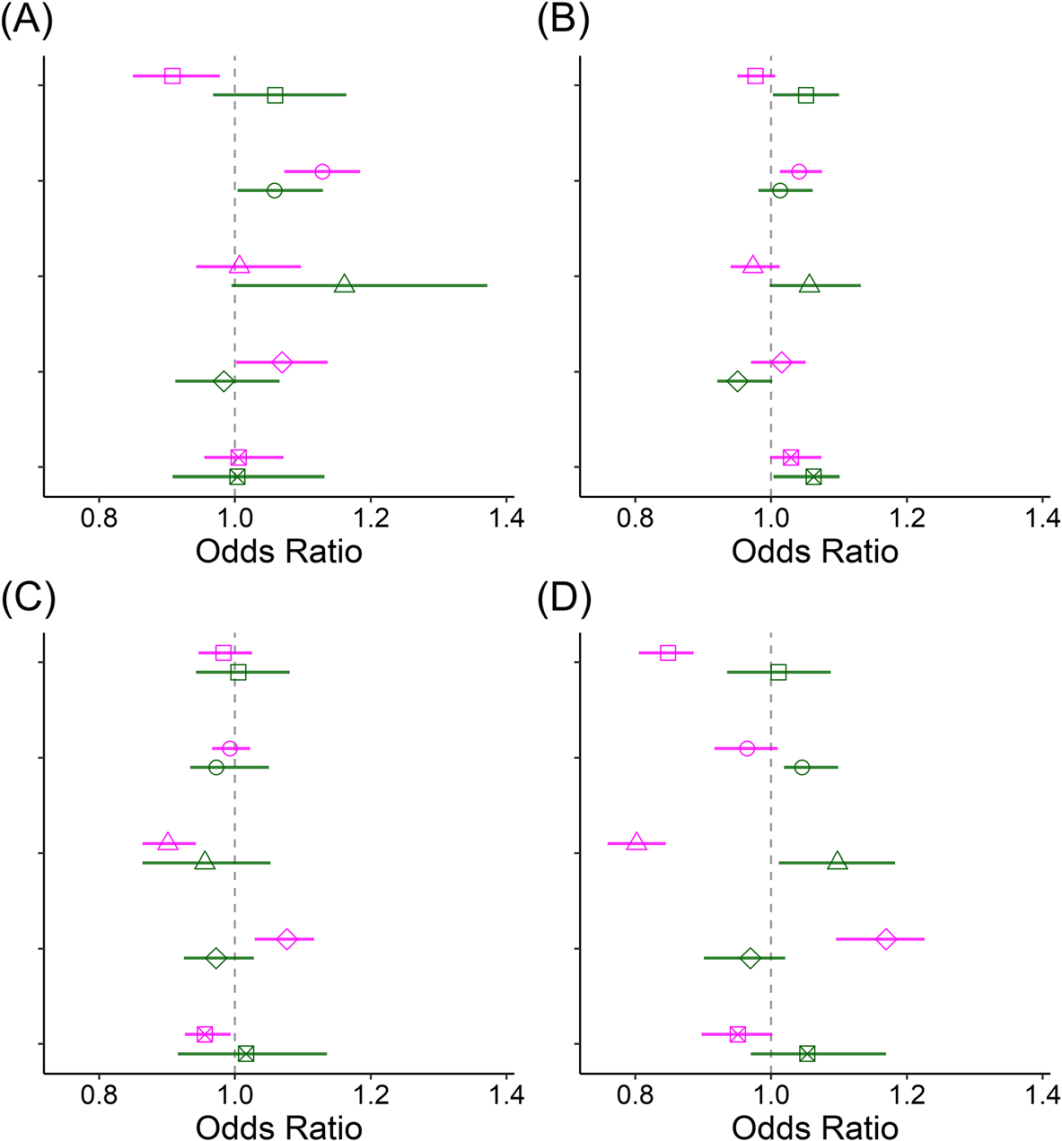
Associations between air pollutant concentrations (□: CO, ○: SO_2_, △: NO_2_, ◇: O_3_, ⊠: PM_10_) and **a** ischemic heart disease (IHD), **b** cerebrovascular disease (CVD), **c** pneumonia (PN), and **d** chronic lower respiratory disease (CLRD) mortality rates in urban (magenta) or rural (green) areas

In the subgroup analysis that divided the 249 districts into capital or non-capital areas (77:172 districts) and into urban or rural areas (168:81 districts), positive associations between SO_2_ concentrations and IHD mortality were consistently observed in all subgroups, while other pollutant-disease pairs showed null or mixed associations (Fig. 2 and Fig. 3). Table 2 summarizes the qualitative associations between disease mortality and air pollutant concentrations in the corresponding subgroup schema. A ‘+’ denote a significant positive association, a ‘–’ to negative, or blank to insignificant. For example, associations between CVD mortality and NO_2_ concentrations exhibited a paradoxical pattern in the subgroup analysis because a significant negative association was found in capital districts but positively associated in non-capital areas. However, null associations were found nationwide and in urban and rural areas. In contrast, significant negative associations were found between NO_2_ concentrations and CLRD mortality nationwide and in capital, non-capital, and urban areas; however, positive associations were found in rural areas.

**Table 2:** Significant associations between mortality rates and air pollutant concentrations nationwide and in capital, non-capital, urban, and rural areas

Ischemic heart disease mortality rate					
Air pollutant	Nationwide	Capital	Non-capital	Urban	Rural
CO				**–**	
SO_2_	**+**	**+**	**+**	**+**	**+**
NO_2_			**+**		
O_3_				**+**	
PM_10_					
Cerebrovascular disease mortality rate					
Air pollutant	Nationwide	Capital	Non-capital	Urban	Rural
CO					**+**
SO_2_	**+**	**+**	**+**	**+**	
NO_2_		**–**	**+**		
O_3_			**–**		
PM_10_	**+**		**+**		**+**
Pneumonia mortality rate					
Air pollutant	Nationwide	Capital	Non-capital	Urban	Rural
CO					
SO_2_	**–**				
NO_2_	**–**	**–**		**–**	
O_3_	**+**			**+**	
PM_10_	**–**			**–**	
Chronic Lower Respiratory Disease mortality rate					
Air pollutant	Nationwide	Capital	Non-capital	Urban	Rural
CO	**–**		**–**	**–**	
SO_2_					**+**
NO_2_	**–**	**–**	**–**	**–**	**+**
O_3_	**+**	**+**		**+**	
PM_10_	**–**				

“+” denotes a positive association, which means increased air pollutant concentrations are associated with higher mortality rates in the study districts

“–” denotes a negative association, which that means the opposite of “+”

Blank denotes null associations

The significance of the associations was determined using 95% confidence intervals

## Discussion

In the nationwide analysis, we found significant positive associations between SO_2_ concentrations and IHD and CVD mortality, PM_10_ concentrations and CVD mortality, and O_3_ concentrations and PN and CLRD mortality, which were consistent with those reported in previous studies [1, 5, 9, 16]; however, direct comparisons of the effect sizes are not appropriate because of differences in the study design, area, and period (Fig. 1). However, significant negative associations between SO_2_ concentrations and PN mortality, NO_2_ concentrations and PN and CLRD mortality, and PM_10_ concentrations and PN and CLRD mortality have not been reported before and are hard to explain intuitively. In subgroup analysis, we consistently found positive associations between SO_2_ concentrations and IHD mortality regardless of the subgrouping schema; hence, we can confidently state that long-term (19 years) exposure to increased SO_2_ concentrations is associated with increased IHD mortality. However, for other disease mortality-air pollutant pairs, it is precarious to conclude that there is a positive or negative association because the beta-regression results differed among the subgroups. Associations between SO_2_ concentrations and CVD mortality were significantly positive nationwide and in capital, non-capital, and urban areas and marginally positive in rural areas; hence, there probably is a positive correlation. For CLRD mortality-NO_2_ pair, the associations are negative in some subgroups and positive in others, which is paradoxical. This paradoxical association patterns among subgroups may imply that there is an important but unidentified confounding factor that was not incorporated in the regression model. For example, in both urban and rural areas, NO_2_ concentrations may be harmful for respiratory health and there may have been an increase medical service usage such as emergency department visits or hospitalization related to CLRD, but accessibility or the quality of medical service may be different in urban and rural areas. In our previous study on the association between air pollution and the incidence and mortality rates of breast cancer, air pollution was positively associated with the incidence rates but not with the mortality rates [13]. This conjecture could be resolved with data on the incidence rates of CLRD per district, which is not presently available.

There are suggested pathways linking long-term exposure to air pollution and cardiopulmonary disease mortality. In a study by Hoek et al., PM_10_ concentrations were associated with a significant increase in blood pressure and induced infection and inflammation in circulatory and respiratory diseases [17]. Hiraiwa et al. have suggested that excess cytokines such as interleukin (IL)-1, IL-6, and tumor necrosis factor (TNF) can induce vascular events in patients with chronic obstructive pulmonary disease via systemic oxidative stress and inflammation in the lung to promote endothelial dysfunction and atherosclerotic plaque rupture, possibly leading to acute cardiac events or stroke [18]. According to Mukae et al., human alveolar macrophages, when exposed to high PM_10_ concentrations, can phagocytose these particles and produce an array of cytokines such as TNFα and IL-1β, which are part of the innate immune response [19]. Hence, long-term exposure to PM_10_ may aggravate premature mortality from CVD and CLRD.

The consistent positive associations between SO_2_ and IHD and CVD mortality that we found agree with previous publications those reported similar associations in short-term. In a systematic review on air pollution and stroke by Shah et al. [20] reported significant positive associations between SO_2_ and mortality and hospital admissions by stroke. Hong et al. [21] found a significant positive association between ischemic stroke mortality and SO_2_ and total suspended particulates in the short term (0–3 lagged days) in Seoul, South Korea. Qian et al. [22] reported significant associations of cardiovascular disease mortality with PM_10_ and SO_2_ in Wuhan, China. Moolgavkar et al. [23] also found out similar associations in Los Angeles, United States. Wichmann et al. [24] reported associations between cardiovascular and cerebrovascular mortality with SO_2_ in Cape Town, South Africa, too. Amancio et al. [25] also found positive associations between short term SO_2_ exposure and circulatory disease and stroke mortality in Brazil with ecological study design. Moreover, Chung et al. [26] found significant positive associations between PM_10_ and SO_2_ and cardioembolic stroke incidence on the basis of the Clinical Research Center for Stroke 5th division centers registry data in South Korea. There are studies suggesting etiological links between SO_2_ exposure and IHD and CVD. Routledge et al. [27] found out that SO_2_ exposure reduce cardiac vagal control, a response that would be expected to increase susceptibility to ventricular arrhythmia. Szyszkowicz et al. [28] suggested that SO_2_-derived acidic compounds may penetrate the brain barrier to mediate abnormal brain neural activity or brain ischemia.

The present study is an ecological analysis rather than an individual-level cohort study nor a case-control study. From the ecological nature of our study arises a limitation. Because the unit of analysis was a district and not an individual, we did not obtain patient-specific information, such as comorbidities, medication, occupational history, and patient-specific exposure to air pollution, that are of considerable importance in cardiopulmonary mortality. Another limitation is the lack of migration history data. We contend that migrations would not significantly impact the current study because only approximately 10% of the population moved between different districts in South Korea between 2003 and 2013 [11, 29]. In addition, there were differences in the density of distributed stations per km^2^(6 times denser in the capital area than in non-capital areas), which could cause potential bias of the daily measurements of the air pollutant concentrations.

## Conclusion

Long-term exposure (19 years) to high SO_2_ concentrations was consistently and significantly associated with a high mortality rate nationwide and in capital and non-capital areas and in urban and rural areas. Associations between other air pollutants and disease-related mortalities need to be investigated in further studies.

## Data Availability

All datasets used during the current study are publicly available via AirKorea (https://www.airkorea.or.kr/) and Korean Statistical Information Service (https://kosis.kr/).

## Abbreviations

IHD: Ischemic heart disease
CVD: Cerebrovascular disease
PN: Pneumonia
CLRD: Chronic lower respiratory disease
CO: Carbon monoxide
SO_2_: Sulfur dioxide
NO_2_: Nitrogen dioxide
O_3_: Ozone
PM_10_: Particulate matter 10 μm or less in diameter
GRDP: Gross regional domestic product per capita
KOSIS: Korean Statistical Information Service
IQR: Interquartile range
OR: Odds Ratio
IL: Interleukin
TNF: Tumor necrosis factor
